# A Green and Blue Monochromatic Light Combination Therapy Reduces Oxidative Stress and Enhances B-Lymphocyte Proliferation through Promoting Melatonin Secretion

**DOI:** 10.1155/2021/5595376

**Published:** 2021-03-19

**Authors:** Yijia Zhang, Zixu Wang, Jing Cao, Yulan Dong, Yaoxing Chen

**Affiliations:** Laboratory of Anatomy of Domestic Animals, College of Veterinary Medicine, China Agricultural University, Haidian, Beijing 100193, China

## Abstract

Artificial illumination may interfere with biological rhythms and distort physiological homeostasis in avian. Our previous study demonstrated that 660 nm red light exacerbates oxidative stress, but a combination of green and blue lights (G→B) can improve the antibody titer in chickens compared with single monochromatic light. Melatonin acts as an antioxidant which is a critical signaling to the coordination between external light stimulation and the cellular response from the body. This study further clarifies the potential role of melatonin in monochromatic light combination-induced bursa B-lymphocyte proliferation in chickens. A total of 192 chicks were exposed to a single monochromatic light (red (R), green (G), blue (B), or white (W) lights) or various monochromatic light combinations (B→G, G→B, and R→B) from P0 to P42. We used qRT-PCR, MTT, western blotting, immunohistochemistry, and Elisa to explore the effect of a combination of monochromatic light on bursa B-lymphocytes and its intracellular signal pathways. With consistency in the upregulation in melatonin level of plasma and antioxidant enzyme ability, we observed increases in organ index, follicle area, lymphocyte density, B-lymphocyte proliferation, PCNA-positive cells, and cyclin D1 expression in bursa of the G→B group compared with other light-treated groups. Melatonin bound to Mel1a and Mel1c and upregulated p-AKT, p-PKC, and p-ERK expression, thereby activating PI3K/AKT and PKC/ERK signaling and inducing B-lymphocyte proliferation. Overall, these findings suggested that melatonin modulates a combination of green and blue light-induced B-lymphocyte proliferation in chickens by reducing oxidative stress and activating the Mel1a/PI3K/AKT and Mel1c/PKC/ERK pathways.

## 1. Introduction

With the widespread application of artificial illumination, light pollution has become a rapidly increasing and global phenomenon. The presence of artificial light at night will disrupt the circadian rhythm of organisms, affect behavioural traits, and distort physiological homeostasis [1]. Birds are sensitive to light because of their highly developed visual systems. In addition to the intensity and photoperiod of light, the wavelength of light also plays an important role in influencing the behaviour, growth, and health of poultry [2]. Our previous laboratory studies showed that 660 nm red light inhibits the growth of chicks and decreases the activities of antioxidant enzymes [3]. In contrast, 560 nm green light enhances muscle growth [4], satellite cell mitotic activity [5], meat quality properties [6], and reduces oxidative stress [3] during the early stage (posthatching (P)0–P26), and 480 nm blue light is more effective during the later stage (P27–P42) [4]. Based on this research, we observed that a combination of green and blue monochromatic light (G→B or B→G) resulted in better productive performance of chickens [7] and produced more antibodies to adapt to the outside environment [8]. The bursa of Fabricius is the central humoral immune organ that is unique to birds and plays an important role in B cell development and antibody production [9]. Our previous study found that a combination of green and blue monochromatic light could promote peripheral blood T and B-lymphocyte proliferation. Simultaneously, the levels of anti-Newcastle disease virus (NDV) and anti-bovine serum albumin (BSA) IgG in G→B group were elevated compared to single monochromatic lights [8]. Thus, the proliferation activity of B-lymphocytes is closely related to the production of antibodies. However, the effects of monochromatic light combinations on B-lymphocyte proliferation and its intracellular mechanisms remain unclear.

Despite a large number of reports on the subject [8], the mechanisms underlying the effects of light wavelengths on the immune response still remain to be explored. Melatonin is a neuroendocrine hormone that regulates immune responses, mitochondrial function, and apoptosis and prevents oxidative stress [10]. A large number of studies have demonstrated the presence of melatonin receptors in a variety of immune cells from various species [11], which explains the immunomodulatory capacity of melatonin administration both in vivo and in vitro. In addition, melatonin is uncommonly effective in attenuating cellular apoptosis and extending cellular longevity via a variety of means: direct free radical scavenging and indirectly by stimulating antioxidant enzymes [12]. Our lab found that green light stimulated pinealocytes and retinal cells, increasing arylalkylamine N-acetyltransferase mRNA levels and melatonin secretion in chickens [13]. Moreover, we found that pinealectomy not only decreased the concentration of melatonin in plasma but also decreased the thymus T-cell proliferation activity [14] and antioxidative capacity [3] of chickens. These findings suggest that melatonin may transmit external light signals to intracellular molecules to regulate the immune level and antioxidative capacity of chickens. Thus, one question arises: does melatonin play a role in promoting monochromatic light combination-induced B-lymphocyte proliferation?

More than 15 different proteins have been proposed to bind melatonin ranging from receptors, enzymes, pore proteins, and transporters to various other proteins [15]. G-protein-coupled receptors are currently the best-characterized melatonin targets and are found in invertebrates and vertebrates. These receptors are classified into three membrane receptors called Mel1a, Mel1b, and Mel1c in chickens [16]. The best-characterized role of melatonin receptors is in the synchronization of biological rhythms, but increasing evidence suggests that melatonin and its receptors regulate a much broader panel of physiological functions, including sleep, pain, and retinal, neuronal, and immune functions [17, 18]. Our previous studies showed that melatonin enhanced green light-induced T-lymphocyte proliferation in chickens via Mel1b and Mel1c receptors [19]. Overall, these results suggest that melatonin plays a key role in regulating cell proliferation and development. However, the downstream signaling pathway triggered by melatonin that promotes cell proliferation depends on the cell state and type [20, 21]. Thus, an investigation into whether melatonin promotes bursa B-lymphocyte proliferation through its membrane receptor and related intracellular signaling pathways is still required.

In this study, we raised chickens under different monochromatic light combinations and explored the extent to which monochromatic light combinations contributed to the development of bursa B-lymphocytes and assessed whether melatonin was involved in this process. Furthermore, we postulated a mechanism for melatonin membrane receptor participation in this regulated process. Additionally, as the poultry industry transitions to antibiotic-free production, there is an urgent need to find economic solutions to improve the immune performance of poultry. If bursa structure, function, and antioxidant level can be influenced by different monochromatic light combinations in its developmental state, this approach can become a potentially valuable and economical approach to maintain body health and improve production efficiency in poultry.

## 2. Materials and Methods

### 2.1. Animals and Treatment

All experimental procedures were approved by the Animal Welfare Committee of the Agricultural Research Organization, China Agricultural University (Approval No. CAU 20171114-2). A total of 192 posthatching day (P0) Arbor Acre male chickens (Beijing Hua du Breeding Co., Beijing, China) were used in this study. The chickens had ad libitum access to feed and water, and the diets were formulated to meet the nutrient recommendations for poultry (NRC, 1994). The temperature in the chicken house was set at 32°C for the first week and then reduced to 30°C in the second week, and the relative humidity was maintained at 60% for the entire period [22].

All chickens were randomly housed in four light-controlled rooms (*n* = 48), and each room contained six separate cells (eight birds per cell) at a density of 11.5 birds/m^2^, and each cell had a set of independent light sources. The birds were exposed to white light (WW, 400–700 nm), red light (RR, 660 nm), green light (GG, 560 nm), or blue light (BB, 480 nm), which was powered by a light-emitting diode (LED) system (Zhongshan Junsheng Lighting Technology Co., Ltd. Zhongshan, China). The light parameters are shown in Table 1. When the chickens were 26 d old, 24 chickens from the GG, 24 chickens from the BB, and 24 chickens from the RR groups were transferred to blue, green, or blue light, at 23 : 00, respectively. The remaining chickens were maintained under the original light color. Therefore, the four light control groups (before P27) were changed into seven light groups until P42. The seven light groups were as follows: WW, RR, GG, BB, G→B, B→G, and R→B.

### 2.2. Sampling

At P42, six chickens were randomly selected from each light treatment group and body weights (g) were recorded. Blood samples were collected from veins and heparinized with 1,000 UI/mL heparin in avian saline, followed by decapitation. After centrifugation at 3000 × g for 15 min, the plasma was decanted and stored at -80°C until melatonin measurement. The whole bursa of Fabricius was removed, and the bursa weight of each bird was measured. The organ index was as follows: organ index = bursa weight/body weight.

### 2.3. Hematoxylin-Eosin (H&E) and Immunohistochemical Staining

Paraffin-embedded tissue was cut into 5 *μ*m-thick sections and then stained using H&E. At least 25 random fields in eight sections of each sample were photographed at 400x magnification with a microscope (BX51, Olympus, Tokyo, Japan). The 10 largest bursa follicles per field and a total of 12 000 bursa follicles (six chickens) per treatment were analyzed.

For immunohistochemical staining, the sections were incubated with primary antibodies (rabbit anti-PCNA, 1 : 500; Abcam, Cambridge, UK) overnight at 4°C. Immunoreactivity was visualized by incubating the tissue sections in 0.01 M PBS containing 0.05% 3,3-diaminobenzidine tetrahydrochloride (DAB, Sigma, St. Louis, MO, USA) and 0.003% hydrogen peroxide for 10 min in the dark. Brown-stained cells indicated a positive reaction to PCNA, and positive cells were counted in 25 random fields from five cross-sections for each sample. The data were analyzed by measuring the integrated optical density (IOD) using Image-pro Plus software (Media Cybernetics, Inc., Rockville, MD, USA) [23].

### 2.4. Lymphocyte Proliferation Assay

Bursa tissues were aseptically removed, gently triturated, and filtered through a tissue sieve (200 mesh per 2.5 cm). Then, a single-cell suspension was prepared with RPMI 1640 medium supplemented with 10% fetal bovine serum (FBS) and antibiotics. Cells were then stimulated with LPS (25 *μ*g/mL, Sigma, St. Louis, MO, USA) and different melatonin concentrations (0, 10, 50, 250, 1000, or 2000 pg/mL; Sigma, St. Louis, MO, USA). Then, the cells were incubated at 37°C with 5% CO_2_ for 44 h. B-lymphocyte proliferation was measured with the methyl thiazolyl tetrazolium (Sigma, St. Louis, MO, USA) assay (MTT). The MTT assay results are expressed as the B-lymphocyte stimulation index, which was calculated for each sample by the OD value (570 nm) for cells with stimulation divided by the OD value (570 nm) for cells without stimulation.

Furthermore, to determine the mechanism of action of melatonin, the cell suspensions of the G→B group were prepared with either 10 *μ*M luzindole (a nonselective antagonist of both Mel1a and Mel1b; Santa Cruz Biotechnology Inc., Dallas, TX, USA), 0.1 *μ*M 4P-PDOT (a selective antagonist of Mel1b; Tocris Bioscience, Bristol, UK), 0.1 *μ*M prazosin (a selective antagonist of Mel1c; Santa Cruz Biotechnology Inc., Dallas, TX, USA), 5 *μ*M Go9863 (a pan-PKC inhibitor, T6313, Topscience, TX, USA), 10 *μ*M PD98059 (a selective inhibitor of MEK-1 that is the upstream kinase of ERK1/2, Tocris Bioscience, MO, USA), 5 *μ*M LY294002 (an inhibitor of PI3-kinase inhibitor, L9908, Sigma, St. Louis, MO, USA), 0.5 *μ*M HY102 (an AKT inhibitor, HY-10249A, MCE, Weehawken, USA), or 0.5 *μ*M TWS119 (a GSK inhibitor, HY10590, MCE, Weehawken, USA) for 30 min before the addition of LPS (25 *μ*g/mL, Sigma, St. Louis, MO, USA) and melatonin (250 pg/mL, Sigma, St. Louis, MO, USA). The suspensions were incubated at 37°C with 5% CO_2_ for 44 h, and the OD value was later determined. Additionally, the cells that were incubated with 10 *μ*M luzindole, 0.1 *μ*M 4P-PDOT, or 0.1 *μ*M prazosin for 30 min before the addition of LPS (25 *μ*g/mL, Sigma, St. Louis, MO, USA) and melatonin (250 pg/mL, Sigma, St. Louis, MO, USA) for 30 min were collected and used to detect the cAMP content.

### 2.5. Enzyme-Linked Immunosorbent Assay

The culture cells or plasma was collected and assayed using a competitive inhibition enzyme-linked immunosorbent assay kit (USCN Life Science Inc., Wuhan, China) for melatonin and cAMP according to the manufacturer's instructions. The data were measured using a microplate reader equipped with a 450 nm filter. Each sample was tested in triplicate.

### 2.6. Real-Time Reverse Transcription-Polymerase Chain Reaction (qRT-PCR)

Total RNA (*n* = 6) was extracted with TRIzol reagent (CW0580A, CoWin Biotech Co., Inc., Beijing, China) according to the manufacturer's instructions. qRT-PCR was performed according to previously described methods [24]. All primers used in the present study are shown in Table 2. The experiments were repeated in triplicate.

### 2.7. Western Blot Analysis

Proteins were extracted from tissues and cells with RIPA lysis buffer (CoWin Biotech Co., Inc., Beijing, China) containing a protease inhibitor (Invitrogen). The protein concentration was determined using a bicinchoninic acid (BCA) kit ( P0012, Beyotime Co., Ltd., Shanghai, China). Equal amounts of protein were loaded on SDS-polyacrylamide gel, transferred onto PVDF membranes, and blocked in 5% skimmed milk for 1 h. Subsequently, the membranes were incubated with the following specific antibodies: anti-Mel1a (1 : 1000; orb11085, Biorbyt, Cambridge, UK), anti-Mel1b (1 : 1000; ab203346, Abcam, Cambridge, UK), anti-Mel1c (1 : 500; sc-18574, Santa Cruz Biotechnology Inc., Dallas, TX, USA), anti-phospho-PKC-PAN (pThr497) antibody (1 : 500, sc-13149, Santa Cruz Biotechnology Inc., Dallas, TX, USA), anti-PKC antibody (1 : 200, sc-13149, Santa Cruz Biotechnology Inc., Dallas, TX, USA), anti-phospho-ERK1/2 antibody or anti-ERK1/2 antibody (1 : 4000, M8159, M5670, Sigma, St. Louis, MO, USA), anti-phosphor-AKT (Ser473) antibody (1 : 500, #4060, CST, Boston, USA), anti-AKT antibody (1 : 500, #9272, CST, Boston, USA), anti-phosphor-GSK-3*β* antibody (1 : 500, PAB10055, Abnova, Taiwan, China), anti-GSK-3*β* antibody (1: 500, 22104-1-AP, Proteintech Group, Inc., Wuhan, China), anti-*β*-catenin antibody (1 : 1000, 51067-2-AP, Proteintech Group, Inc., Wuhan, China), anti-cyclin D1 antibody (1 : 200, abx100482, Abbexa, Cambridge, UK), or anti-*β*-actin (1 : 4000; CoWin Biotech Co., Inc., Beijing, China) overnight at 4°C. The membranes were washed with tris-buffered saline tween (TBST) and incubated with horseradish peroxidase- (HRP-) conjugated goat anti-mouse/rabbit antibody (1 : 8000; CoWin Biotech Co., Inc. Beijing, China). The bands obtained in the blots were scanned and analyzed by measurement of the IOD using ImageJ software (version 4.0.2; Scion Corp., Frederick, MD, USA). The values of the target bands were normalized to the corresponding *β*-actin values. The results were obtained from three separate experiments.

### 2.8. Measurements of Antioxidant Activity and Lipid Peroxidation

Portions of the bursa (*n* = 6) were rapidly homogenized, and clarified lysates were obtained by centrifugation (200 g for 10 minutes) at 4°C. The tissue extracts were stored at −80°C for antioxidant activity analysis. Five commercial kits (Beyotime Co., Ltd., Shanghai, China) were used to assay the activities of superoxide dismutase (SOD), catalase (CAT) and glutathione peroxidase (GSH-Px), the total antioxidant capability (T-AOC), and the malondialdehyde (MDA) content using colorimetric methods. Each sample was assayed three times.

### 2.9. Data Analysis

All data are presented as the mean ± standard error of the mean (SEM) and analyzed by one-way ANOVA using SPSS 25.0 software (SPSS, Chicago, IL, USA). Results were considered statistically significant when the *p* value was <0.05. Correlation analysis, expressed as Pearson's coefficient, was performed to determine the possible linear relationship between B-lymphocyte stimulation index under different light colors and melatonin content in the plasma, as well as B-lymphocyte stimulation index under different light colors and protein expressions of Mel1a, Mel1b, and Mel1c, respectively.

## 3. Results

### 3.1. Effects of Monochromatic Light Combinations on the Morphological Change of the Bursa

**Figure 1:**
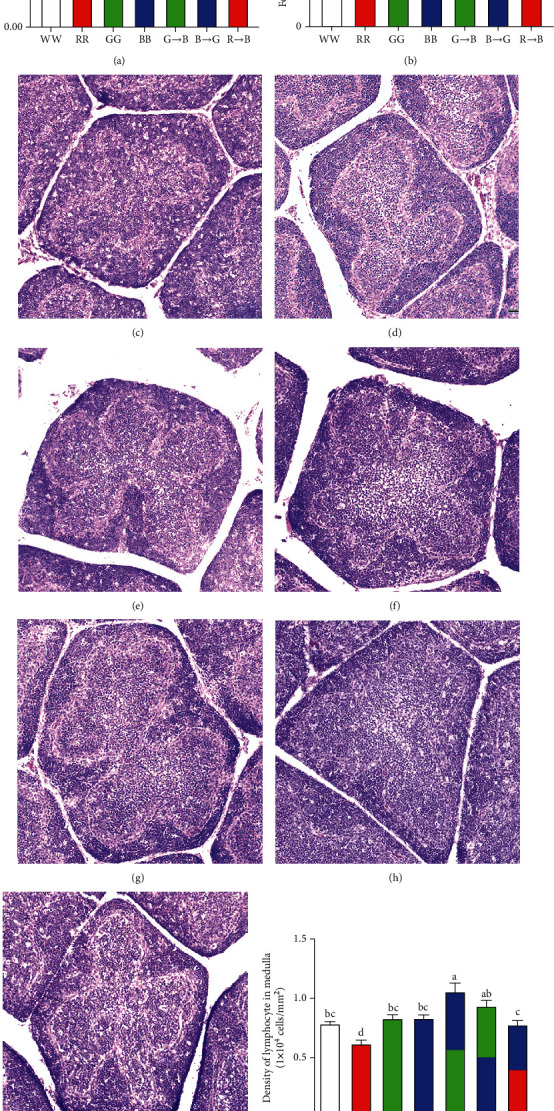
Effects of monochromatic light combinations on the bursa index (a), area of bursa follicle (b–i), and the density of bursa lymphocytes in the medulla (j) (scale bar = 100 *μ*m). WW: white light; RR: red light; GG: green light; BB: blue light; G→B: green light and blue light combination; B→G: blue light and green light combination; R→B: red light and blue light combination. The results are presented as the means ± SEM. Values with no common letters are significantly different (*p* < 0.05) from each other.

To investigate whether monochromatic light combinations promoted bursa development, we examined the morphological changes in the bursa. The organ index is shown in Figure 1(a). At P42, the organ index of G→B was the highest and was significantly larger by 27.61–95.45% (*p* = 0.001-0.038) than that of WW, RR, GG, BB, and R→B, but no significant difference was found between G→B and B→G (*p* = 0.075). The H&E staining results showed that the bursa of the G→B had the largest follicle area and the highest lymphocyte density in the medulla compared to that of WW, RR, GG, BB, and R→B, respectively (Figures 1(b)–1(j)). However, there was no significant difference between G→B and B→G (*p* = 0.090-0.747). In contrast, the organ index, follicle area, and lymphocyte density in the medulla of RR were the lowest.

### 3.2. Effects of Monochromatic Light Combinations on PCNA, Cyclin D1Expression, and B-Lymphocyte Proliferation in the Bursa

**Figure 2:**
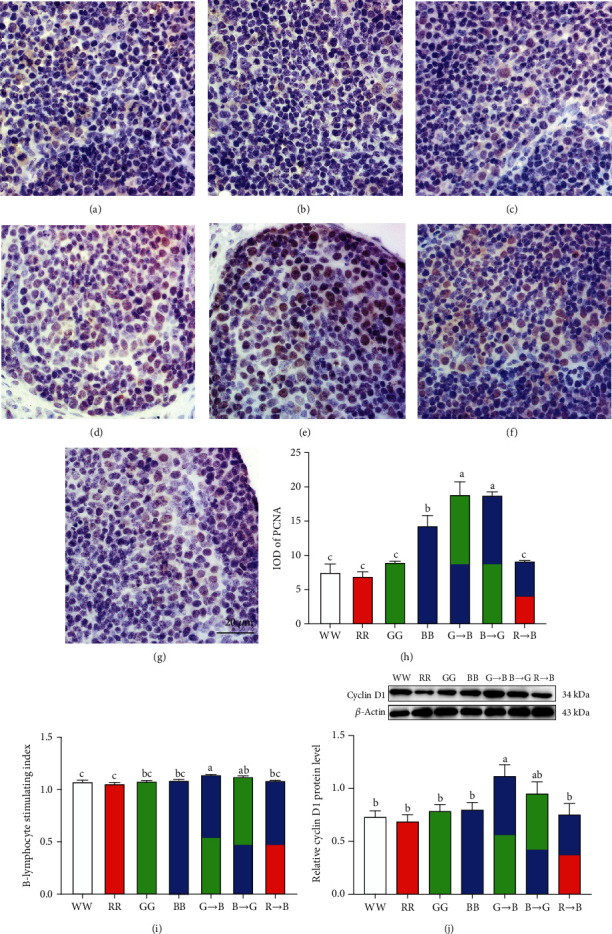
Effects of monochromatic light combinations on immunohistochemical staining of PCNA in the bursa (a–h) (scale bar = 50 *μ*m), bursa B-lymphocyte proliferation in response to LPS (i), and cyclin D1 protein expression (j). WW: white light; RR: red light; GG: green light; BB: blue light; G→B: green light and blue light combination; B→G: blue light and green light combination; R→B: red light and blue light combination. The results are presented as the means ± SEM. Values with no common letters are significantly different (*p* < 0.05) from each other.

Next, we assessed whether a combination of monochromatic light could affect bursa B-lymphocyte proliferation in vivo and in vitro. In vivo, B-lymphocyte proliferation was detected by PCNA immunohistochemistry. The PCNA-positive cells were scattered in the bursa medulla and cortex. As shown in Figures 2(a)–2(h), the IOD of PCNA-positive cells was higher in G→B than in WW, RR, GG, BB, and R→B (31.72–170.44%, *p* = 0.001-0.025). Similarly, the proliferation of bursa B-lymphocytes in response to LPS treatment was highest in the G→B group at P42 (Figure 2(i)). There was no significant difference between G→B and B→G (*p* = 0.353), but G→B was higher than B→G by 1.29%. Additionally, we tested the protein level of cyclin D1, which plays an important role in the G1 phase progression of the cell cycle in proliferating cells. As shown in Figure 2(j), cyclin D1 expression was higher in G→B than in WW, RR, GG, BB, and R→B (38.78–61.31%, *p* = 0.009-0.028). However, there was no significant difference between G→B and B→G (*p* = 0.225). In contrast to the G→B, 660 nm red light evoked a substantial reduction of PCNA-positive cells, cyclin D1 expression, and B-lymphocyte proliferation.

### 3.3. Effects of Monochromatic Light Combinations on Plasma Melatonin Level and Bursa Antioxidant Status

**Figure 3:**
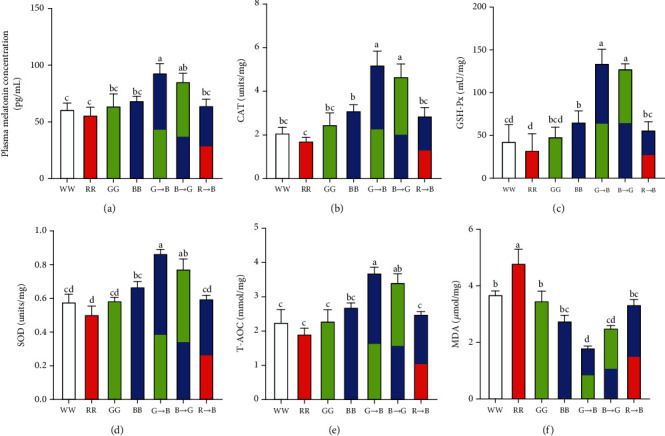
Effects of monochromatic light combinations on plasma melatonin concentration (a), CAT (b), GSH-Px (c), SOD (d), T-AOC (e), and MDA (f) concentrations in the bursa. WW: white light; RR: red light; GG: green light; BB: blue light; G→B: green light and blue light combination; B→G: blue light and green light combination; R→B: red light and blue light combination. The results are presented as the means ± SEM. Values with no common letters are significantly different (*p* < 0.05) from each other.

The plasma melatonin levels of chickens after exposure to various monochromatic lights are shown in Figure 3(a). By 42 d, the chickens that were exposed to G→B showed significantly higher levels of circulatory melatonin compared to chickens reared under WW, RR, GG, and R→B (*p* = 0.005-0.045), whereas a significant decrease in the plasma melatonin level was observed in chickens raised under 660 nm red light. There was no significant difference between G→B and B→G (*p* = 0.502), but G→B was higher than that of B→G by 8.89%. Additionally, there was a strong correlation between the melatonin concentration in the plasma and the stimulation index of B-lymphocytes (*r* = 0.9674, *p* ≤ 0.001).

Five antioxidant parameters, including antioxidant enzymes (CAT, GSH-Px, and SOD), T-AOC and MDA, were assayed in bursa. As shown in Figures 3(b)–3(e), the antioxidant enzymes and T-AOC were higher in G→B than in WW, RR, GG, BB, and R→B 66.31–199.40% (CAT, *p* = 0.001-0.045), 67.83-139.55% (GSH-Px, *p* = 0.001-0.031), 29.85-72.28% (SOD, *p* = 0.001-0.010), and 37.04-92.21% (T-AOC, *p* = 0.001-0.017) in the bursa. In addition, we observed a significant decrease in MDA levels, an end product of lipid peroxidation, in the chicken bursa under G→B (Figure 3(f)). However, the MDA content in RR was significantly increased by 29.73-164.22% (*p* = 0.001-0.016) than in WW, RR, GG, BB, G→B, and B→G. Pearson's coefficient showed that there was a strong correlation between the melatonin concentration in the plasma and CAT (*r* = 0.9649, *p* ≤ 0.001), GSH-Px (*r* = 0.9392, *p* = 0.002), SOD (*r* = 0.9757, *p* ≤ 0.001), T-AOC (*r* = 0.9701, *p* ≤ 0.001), and MDA (*r* = −0.9562, *p* ≤ 0.001).

### 3.4. Effects of Exogenous Melatonin on B-Lymphocyte Proliferation In Vitro

**Figure 4:**
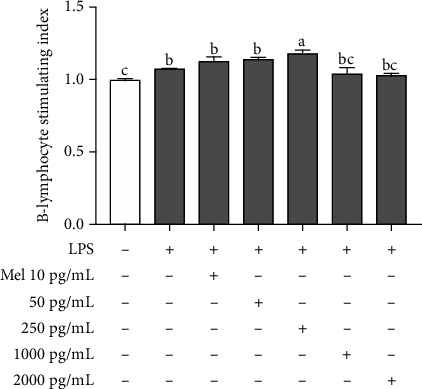
Effects of monochromatic light combinations on exogenous melatonin on B-lymphocyte proliferation in the bursa in response to LPS in the G→B group (a). The results are presented as the means ± SEM. Values with no common letters are significantly different (*p* < 0.05) from each other.

To further verify whether exogenous melatonin promoted the proliferation of B-lymphocytes in vitro, we used an MTT assay to determine the effect of 0 pg/mL to 2 000 pg/mL of melatonin on B-lymphocyte proliferation after 44 h of culture. The results of MTT assay were expressed in the form of B-lymphocyte stimulation index. As shown in Figure 4a, when the concentration of exogenous melatonin was ≤250 pg/mL, there was a strong correlation to strengthen the statement of melatonin enhanced lymphocyte proliferation in a dose-dependent manner (*r* = 0.9994, *p* = 0.022). According to the results of the MTT assay, we chose 250 pg/mL of melatonin for the follow-up experiments.

### 3.5. Effects of Monochromatic Light Combinations on Melatonin Receptor Expression in the Bursa

**Figure 5:**
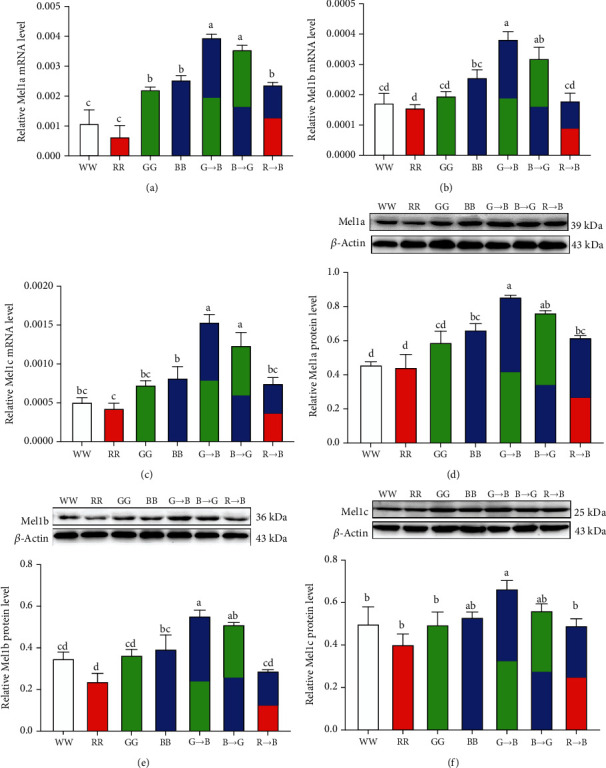
Effects of monochromatic light combinations on *Mel1a* mRNA expression (a), *Mel1b* mRNA expression (b), *Mel1c* mRNA expression (c), Mel1a protein expression (d), Mel1b protein expression (e), and Mel1c protein expression (f) in the bursa at P42. Mel1a: melatonin receptor subtype 1a; Mel1b: melatonin receptor subtype 1b; Mel1c: melatonin receptor subtype 1c; WW: white light; RR: red light; GG: green light; BB: blue light; G→B: green light and blue light combination; B→G: blue light and green light combination; R→B: red light and blue light combination. The results are presented as the means ± SEM. Values with no common letters are significantly different (*p* < 0.05) from each other.

The classical melatonin signaling was induced by binding to its membrane receptors (Mel1a, Mel1b, and Mel1c), then activated melatonin membrane receptors initiating downstream intracellular signaling pathways. To investigate the effects of a combination of monochromatic light on the melatonin membrane receptors, we measured the expression levels of *Mel1a*, *Mel1b*, and *Mel1c* mRNA and protein. As shown in Figures 5(a)–5(c), G→B significantly upregulated *Mel1a*, *Mel1b*, and *Mel1c* expression, while RR suppressed the *Mel1a*, *Mel1b*, and *Mel1c* expression at the mRNA levels. However, there were no significant differences between G→B and B→G (*p* = 0.089-0.315). Similar results were seen in western blot analysis (Figures 5(d)–5(f)), although no differences were found among G→B and B→G (*p* = 0.182-0.473). Additionally, a strong correlation between B-lymphocyte proliferation and the protein expression of Mel1a (*r* = 0.9111, *p* = 0.004), Mel1b (*r* = 0.9307, *p* = 0.002), and Mel1c (*r* = 0.8949, *p* = 0.007) was noted.

### 3.6. Effects of Monochromatic Light Combinations on p-AKT, p-PKC, p-ERK, p-GSK-3*β*, and *β*-Catenin Protein Expression in the Bursa

**Figure 6:**
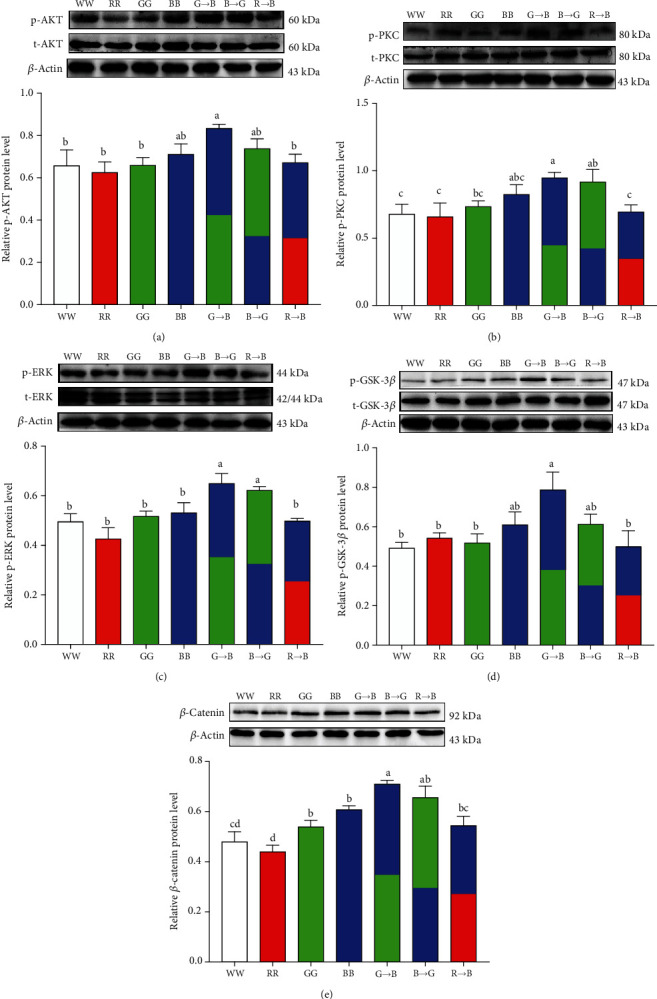
Effects of monochromatic light combinations on p-AKT/total-AKT ratio (a), p-PKC/total-PKC ratio (b), p-ERK/total-ERK ratio (c), p-GSK-3*β*/total-GSK-3*β* ratio (d), and *β*-catenin protein level (e) in bursa at P42. WW: white light; RR: red light; GG: green light; BB: blue light; G→B: green light and blue light combination; B→G: blue light and green light combination; R→B: red light and blue light combination. The results are presented as the means ± SEM. Values with no common letters are significantly different (*p* < 0.05) from each other. p-AKT: phosphorylated AKT; p-PKC: phosphorylated PKC; p-ERK: phosphorylated ERK; p-GSK-3*β*: phosphorylated GSK-3*β*.

To determine the molecular mechanisms underlying the effects of a combination of monochromatic light-induced B-lymphocyte proliferation, we examined the expression of PI3K/AKT signaling and PKC/ERK signaling-related proteins in chicken bursa under WW, RR, GG, BB, G→B, B→G, and R→B. Western blot analysis revealed that p-AKT, p-PKC, p-ERK, p-GSK-3*β*, and *β*-catenin proteins were significantly upregulated in G→B (Figures 6(a)–6(e)), but no significant difference was found between G→B and B→G (*p* = 0.054-0.772).

### 3.7. Effects of Melatonin Receptors on the Monochromatic Light Combination-Induced Bursa B-Lymphocyte Proliferation

**Figure 7:**
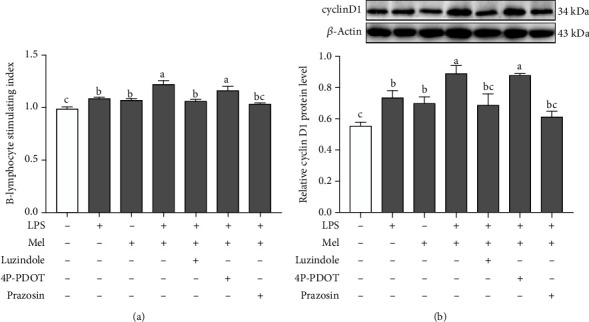
Effects of different melatonin receptor antagonists on B-lymphocyte proliferation (a) and cyclin D1 protein (b) in the G→B group. Luzindole is a nonselective Mel1a/Mel1b antagonist; 4P-PDOT is a selective Mel1b antagonist; prazosin is a selective Mel1c antagonist. Values with no common letters are significantly different (*p* < 0.05) from each other. 4P-PDOT: 4-phenyl-2-propionamideotetralin; LPS: lipopolysaccharide; Mel: melatonin; SI: B-lymphocyte stimulation index.

To determine the involvement of melatonin receptors on B-lymphocyte proliferation, primary cultures were pretreated with luzindole (a nonselective antagonist of both Mel1a and Mel1b), 4P-PDOT (a selective antagonist of Mel1b), and prazosin (a selective antagonist of Mel1c). As shown in Figures 7(a) and 7(b), we found that pretreatment of B-lymphocytes with luzindole or prazosin in response to LPS + melatonin significantly decreased the B-lymphocyte stimulating index and cyclin D1 protein expression by 13.01–15.93% (*p* ≤ 0.001) and 22.93–31.29% (*p* = 0.001-0.007) compared with the LPS + melatonin-cotreated group, respectively. However, 4P-PDOT (0.1 *μ*M), which was coincubated with LPS and melatonin, showed no statistical significance when compared with the LPS + melatonin-cotreated group (*p* = 0.120).

### 3.8. Mel1a/Gi/PI3K/AKT and Mel1c/PKC/ERK Signaling Pathways Are Involved in a Melatonin-Mediated Monochromatic Light Combination-Induced B-Lymphocyte Proliferation

**Figure 8:**
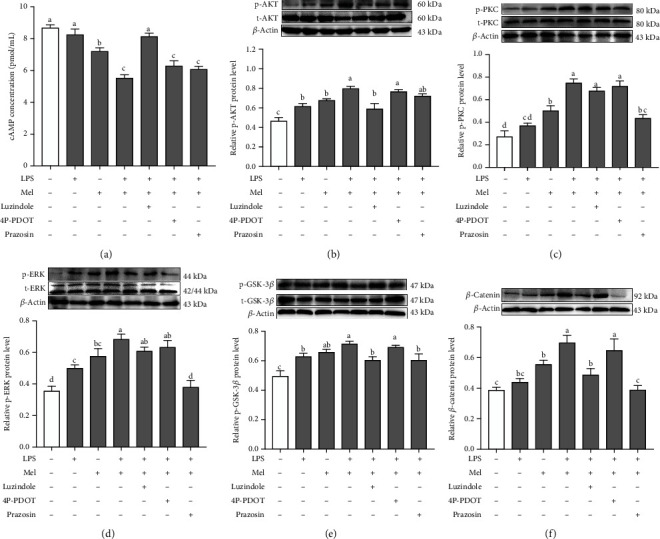
Effects of different melatonin receptor antagonists on the level of intracellular cAMP (a), p-AKT/total-AKT ratio (b), p-PKC/total-PKC ratio (c), p-ERK/total-ERK ratio (d), p-GSK-3*β*/total-GSK-3*β* ratio (e), and *β*-catenin protein level (f) in the G→B group. Luzindole is a nonselective Mel1a/Mel1b antagonist; 4P-PDOT is a selective Mel1b antagonist; prazosin is a selective Mel1c antagonist. Values with no common letters are significantly different (*p* < 0.05) from each other. 4P-PDOT: 4-phenyl-2-propionamideotetralin; LPS: lipopolysaccharide; Mel: melatonin. p-AKT: phosphorylated AKT; p-PKC: phosphorylated PKC; p-ERK: phosphorylated ERK; p-GSK-3*β*: phosphorylated GSK-3*β*.

Melatonin receptors are typically coupled to Gi, Gq, or Gs proteins. To identify which specific G proteins coupled to Mel1a or Mel1c for the relay of melatonin to downstream activation in B-lymphocytes, we isolated B-lymphocytes from G→B chicken bursa and treated them with melatonin, LPS, and melatonin receptor antagonist either alone or together. Then, cAMP production was determined by ELISA. As shown in Figure 8(a), the melatonin + LPS-cotreated group showed significantly decreased cAMP levels (36.10%) compared to that of the control group (*p* ≤ 0.001), and this response was prevented by luzindole and not affected by 4P-PDOT or prazosin. Taken together, these data indicated that Gi might couple to Mel1a and be involved in B-lymphocyte proliferation in G→B chickens. Notably, pretreatment of B cells with Mel1c antagonists (prazosin) before melatonin addition did not affect melatonin-induced inhibition of cAMP levels, indicating that Mel1c might not depend on Gi or Gs to activate B-lymphocyte proliferation.

To characterize the respective roles of melatonin receptors in PI3K/AKT and PKC/ERK pathway, we pretreated cells with melatonin membrane receptor antagonists. As shown in Figure 8(b), the observed melatonin-induced upregulation in the ratio of p-AKT/total-AKT was abrogated by luzindole and not affected by 4P-PDOT or prazosin. Additionally, melatonin-induced upregulation of p-PKC and p-ERK1/2 protein expression was abrogated by the Mel1c antagonist and not affected by 4P-PDOT or luzindole (Figures 8(c) and 8(d)). Unexpectedly, luzindole and prazosin both significantly inhibited the p-GSK-3*β*/total-GSK-3*β* ratio (*p* = 0.014) and *β*-catenin protein expression (*p* = 0.001-0.004) compared with the LPS + melatonin cotreatment (Figures 8(e) and 8(f)).

### 3.9. Mel1a-Mediated PI3K/AKT Signaling Pathway and Mel1c-Mediated PKC/ERK Signaling Pathway Have a Cooperative Action in Promoting Melatonin-Induced B-Lymphocyte Proliferation

**Figure 9:**
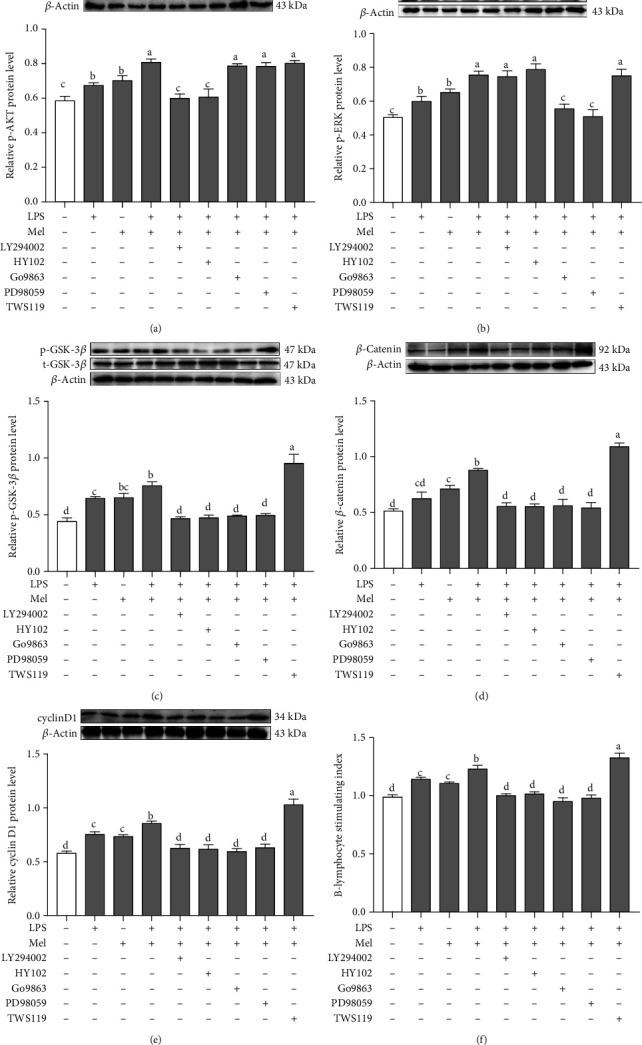
Effects of PI3K inhibitor, AKT inhibitor, PKC inhibitor, ERK1/2 inhibitor, and GSK-3*β* inhibitor on p-AKT/total-AKT ratio (a), p-ERK/total-ERK ratio (b), p-GSK-3*β*/total-GSK-3*β* ratio (c), *β*-catenin protein level (d), cyclin D1 protein level (e), and B-lymphocyte proliferation stimulating index (f). LY294002 is a PI3-kinase inhibitor; HY102 is an AKT inhibitor; Go9863 is a PKC inhibitor; PD98059 is an ERK inhibitor; TWS119 is a GSK inhibitor. Values with no common letters are significantly different (*p* < 0.05) from each other. LPS: lipopolysaccharide; Mel: melatonin. p-AKT: phosphorylated AKT; p-PKC: phosphorylated PKC; p-ERK1/2: phosphorylated ERK1/2; p-GSK-3*β*: phosphorylated GSK-3*β*.

To further confirm that the PI3K/AKT pathway was involved in melatonin-induced B-lymphocyte proliferation, we treated cells with LY294002, a PI3K inhibitor, and HY102, an AKT inhibitor. We found that the treatment of isolated B-lymphocytes with LPS + melatonin for 30 min markedly elevated the ratios of p-AKT/total-AKT (*p* ≤ 0.001) compared with the control cells. However, LY294002 and HY102 significantly abrogated the melatonin induced-upregulation of the p-AKT/total-AKT ratio (*p* ≤ 0.001) (Figure 9(a)) and inhibited B-lymphocyte proliferation (*p* ≤ 0.001) compared with the LPS + melatonin-cotreated group (Figure 9(f)). In addition, we blocked the activity of PKC and ERK using the specific inhibitors Go9863 and PD98059 to determine whether the Mel1c-mediated B-lymphocyte proliferation is PKC/ERK-dependent or independent. The western blot assays and MTT assay showed that Go9863 or PD98059 significantly decreased the ratio of p-ERK/total-ERK (*p* ≤ 0.001) and inhibited B-lymphocyte proliferation (*p* ≤ 0.001) compared with the LPS + melatonin-cotreated group (Figures 9(b) and 9(f)). To better understand the different intracellular signal dependency of B-lymphocytes by Mel1a and Mel1c, we determined the downstream pathways involved in PI3K/AKT and PKC/ERK. It was previously reported that GSK-3*β*/*β*-catenin pathways are important candidates as downstream mediators of AKT protein. As shown in Figures 9(c) and 9(d), the results showed that LPS + melatonin cotreatment had markedly elevated p-GSK-3*β*/total-GSK-3*β* ratio (*p* ≤ 0.001) and the protein level of *β*-catenin compared with the control cells (*p* ≤ 0.001). Additionally, the phosphorylation of GSK-3*β* and *β*-catenin evoked by treatment with melatonin was markedly inhibited by treatment with LY294002 (*p* ≤ 0.001), or HY102 (*p* ≤ 0.001). Consistent with this, the promoting effect of melatonin on cyclin D1 protein expression and B-lymphocyte proliferation was significantly strengthened by the GSK inhibitor TWS119 (*p* = 0.001-0.012) (Figures 9(e) and 9(f)). Unexpectedly, Go9863 and PD98059 both significantly inhibited the p-GSK-3*β*/total-GSK-3*β* ratio (*p* ≤ 0.001), *β*-catenin (*p* ≤ 0.001), and cyclin D1 (*p* ≤ 0.001) protein expression compared with the LPS + melatonin cotreatment (Figures 9(c)–9(e)).

## 4. Discussion

### 4.1. Effects of Monochromatic Light Combinations on B-Lymphocyte Proliferation in the Bursa

As a key cell type mediating humoral adaptive immunity, B-lymphocytes have first been described in chickens as antibody-producing cells [25]. Consistent with this essential function, B-lymphocyte development and maturation can be affected by several environmental factors, such as the caging environment [26] and heat stress [27]. Thus, we constructed a light-color conversion system throughout the growth of chickens to explore the effect of monochromatic light combinations on B-lymphocyte proliferation and bursa development and assess whether melatonin was involved in this process. In present study, a combination of green and blue monochromatic light increased melatonin secretion. Then, melatonin exerted its proliferation-inducing effect on LPS-stimulated B-lymphocytes by reducing oxidative stress and activating Mel1a/PI3K/AKT and Mel1c/PKC/ERK pathways in bursa.

In this study, we found that compared with other light treatments, G→B and B→G significantly increased the organ index, area of the bursa follicle, and density of bursa lymphocytes in the medulla. At present, there is a scoring scale to evaluate the development of the bursa of Fabricius. They found that organ index, histopathology scoring, and morphometric analysis of the total follicle area were associated with the extent of bursa development [28] and increased index of immune organs or histologic changes, suggesting enhanced immune function and ability to resist various infections, diseases, and stress [29]. Based on these results, we demonstrated that G→B and B→G could better promote the bursa of Fabricius development and RR reverses this stimulatory effect on the bursa development. Our results were also supported by previous studies that used a meta-analysis to establish light spectral models of chickens and verify the shift to green-blue of combined LED lights that could produce the optimized production performance [30]. However, the effects of light wavelengths on growth and development are also related to the species and photoperiod. In Yangzhou geese, white or red monochromatic lights, when imposing a long photoperiod of 11 h daily, could result in greater egg-laying performance [31].

In the present study, we demonstrated that G→B and B→G could promote B-lymphocyte proliferation and RR has the opposite effects. PCNA is an indicator for evaluating the state of cell proliferation, and cyclin D1 is considered a proliferation marker. The upregulation of PCNA and cyclin D1 indicated an increase in cell proliferation. A similar observation has documented that a combination of monochromatic light could effectively enhance the peripheral blood T-lymphocyte proliferation of chickens compared to single monochromatic lights [8]. These results revealed that a combination of green and blue monochromatic light may increase mitogenic activity and B-lymphocyte proliferation, which would result in the enhancement of B cell antibody production.

### 4.2. Effects of Melatonin on Monochromatic Light Combination-Induced B-Lymphocyte Proliferation

Along with increased B-lymphocyte proliferation, G→B increased the plasma melatonin concentration in chickens. Similar observations have been documented in the early stages of chicken, which have been reported to promote the secretion of melatonin by enhancing pinealocytes to express melatonin key synthetase AA-NAT mRNA, upregulating the expression of positive clock genes, and downregulating the expression of negative clock genes [32]. Interestingly, Pearson's correlation analysis showed that melatonin was strongly positively correlated with B-lymphocyte proliferation. It was previously reported that the physiological levels of melatonin in chickens are in 60-250 pg/mL (0.26∼1.08 nmol/L) in the blood [33]. In the present study, our results showed that exogenous melatonin at a level of 250 pg/mL stimulated B-lymphocyte proliferation in the bursa. Consistent with our findings, Luo et al. found that melatonin has an activation effect on mouse peripheral blood T/B cells in mice [34]. However, when the exogenous melatonin concentration > 250 pg/mL, the B-lymphocyte stimulating index was decreased. In fact, when the melatonin exceeds the physiological concentration, it will inhibit cell activity. Gao et al. found that treatment with melatonin at the dose from 0.125 mmol/L to 1 mmol/L significantly inhibited cell viability in SW620 and LOVO cells, respectively [35]. Wang et al. found melatonin at pharmacological concentrations (1 mmol/L) significantly suppressed cell proliferation and induced apoptosis in human breast cancer cells [36].

Melatonin, as a key endogenous factor in limiting free radical damage, was found to efficiently improve the reductive potential of tissues and fluids. In the present study, we found that a green and blue monochromatic light combination illumination significantly increased the activities of GSH-Px, SOD, CAT, and T-AOC, resulting in the reduction of the MDA content in the bursa of chickens as compared with other monochromatic lights. Moreover, Pearson's correlation analysis showed that melatonin was strongly positively correlated with the activity of GSH-Px, SOD, CAT, and T-AOC whereas negatively correlated with the content of MDA. A previous report indicated that the total antioxidant capacity of human blood was found to be positively correlated with endogenous melatonin [37], which agreed with our findings. These results suggested that not only pharmacological levels of melatonin but also physiological concentration could provide protection against free radical damage. In addition, melatonin, an amphiphilic compound, crossed easily through biological membranes with high antioxidative power [38] and is involved in the inhibition of apoptosis and repair of DNA damage [39]. Overall, our results strongly indicated that melatonin plays an important role in regulating antioxidant enzyme capacity, preventing oxidative stress during the monochromatic light combination-induced B-lymphocyte proliferation in response to LPS.

### 4.3. Role of Melatonin Receptors in Melatonin-Mediated Monochromatic Light Combination-Induced B-Lymphocyte Proliferation

It is well known that melatonin exhibits its immunomodulatory effects via specific receptors [40]. In the present study, we demonstrated that three melatonin receptor subtypes (Mel1a, Mel1b, and Mel1c) were expressed in the bursa, but Mel1b protein expression was markedly lower than Mel1c and Mel1a expression. However, other researchers found that the green light-induced mRNA level of *Mel1a* was lower than that of *Mel1b* and *Mel1c* in the spleen of chickens, suggesting a different role of melatonin membrane receptors in immunomodulation may be related to different cellular situations and various cell types [41]. In vitro experiments, we observed that melatonin-induced B-lymphocyte proliferation was mediated by Mel1a and Mel1c, but not Mel1b. These findings suggested that the effects of melatonin on Jurkat T-lymphocytes seem to be mediated by Mel1a [42]. Ahmad et al. found the possible involvement of Mel1a in regulating splenocyte proliferation in the seasonally breeding tropical rodent [43] supported our speculation. Although Mel1a and Mel1b receptors have a 60% homology, these two melatonin receptors appear to play different roles in mediating immunity. Jockers et al. reported that the Mel1a receptor mainly plays a role in the acquired immune response and the Mel1b receptor was the target for innate immune responses [44]. Additionally, recent research determined that Mel1b plays an important signaling role in mediating melatonin to promote bone marrow stem cell differentiation and bone formation [45]. Thus, we speculated that Mel1b may promote a green and blue combination-induced bursa structure and function development and maturation. Overall, these findings implied that Mel1a, Mel1b, and Mel1c may be involved in a combination of monochromatic light-induced B-lymphocyte proliferation and bursal development, but they may play different roles in this process.

### 4.4. Intracellular Signaling Pathways of Melatonin Receptor-Mediated Monochromatic Light Combination-Induced B-Lymphocyte Proliferation

In this study, we found that melatonin induced a decrease in the intracellular cAMP level in response to LPS, which was scarcely influenced by luzindole and not blocked by 4P-PDOT or prazosin. The descent of cAMP in response to melatonin + LPS treatment might occur because of Gi coupling to Mel1a. This result is consistent with the previous report that Mel1a response is Gi-dependent in NS-1 cells [46], Chinese hamster ovary cells [47], and human embryonic kidney cells [48]. Over the years, AKT/GSK-3*β*/*β*-catenin signaling is essential for B cell survival and development in the bursa [49]. In our study, we found that G→B and B→G significantly promoted AKT, GSK-3*β*, and *β*-catenin phosphorylation compared with WW, RR, GG, BB, and R→B. Melatonin-induced AKT and *β*-catenin activation were blocked by luzindole. The trends for AKT activation and *β*-catenin stabilization under monochromatic light stimulation were consistent with the index of B-lymphocyte stimulation, implying the AKT, GSK-3*β*, and *β*-catenin cascades may participate in a combination of monochromatic light-induced Mel1a-mediated B-lymphocyte proliferation. Consistent with our results, in pluripotent stem cells, melatonin-induced neural differentiation involves the PI3K/AKT pathway and is blocked by luzindole [50]. However, the repertoire of signaling pathways modulated by melatonin receptors is highly cell type-specific. In breast cancer models, melatonin has been reported to involve Mel1a to display antitumoural properties through inhibition of AKT signaling [51]. These results suggested that melatonin activated Gi/PI3K/AKT/GSK-3*β*/*β*-catenin through Mel1a in LPS-stimulated B-lymphocyte proliferation.

In addition, we also observed that G→B and B→G significantly promoted the protein expression of p-PKC and p-ERK, and Mel1c may mediate a green and blue monochromatic light combination stimulated B-lymphocyte proliferation through the intracellular PKC/ERK signaling pathway. Consistent with our findings, Ning et al. reported that melatonin promoted *IGF-1* mRNA expression through the Mel1c-mediated PKC/ERK pathway in chick liver cells [52]. Interestingly, ERK is considered a signaling hub with many different input and output pathways with multiple crosstalks [53]. In our study, pretreatment with Go9863 or PD98059 not only resulted in inhibition of ERK activation but also led to a decrease of GSK-3*β* (Ser 9) phosphorylation and downregulation of *β*-catenin in B-lymphocytes. This result suggested that melatonin-induced *β*-catenin upregulation depends on the common regulation of the PI3K/AKT and PKC/ERK pathways. This suggestion was strengthened by results from a series of studies suggesting there is a direct interaction between ERK and GSK-3*β* in osteoblastic cells [54], HepG2 cells [55], and RAW264.7 cells [56]. Taken together, our findings provided a strong evidence that the Mel1a-mediated PI3K/AKT signaling pathway and the Mel1c-mediated PKC/ERK signaling pathway have a cooperative action in promoting melatonin-induced B-lymphocyte proliferation.

## 5. Conclusion

**Figure 10:**
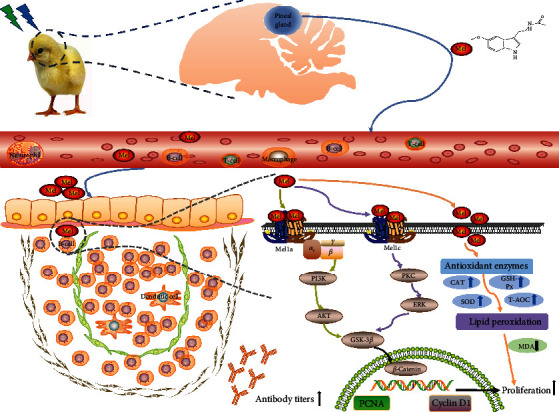
Hypothetical diagram of how melatonin modulates a combination of green and blue light-induced B-lymphocyte proliferation in chickens. A combination of green and blue monochromatic light increased melatonin secretion. Then, melatonin reduced oxidative stress and activated Mel1a/PI3K/AKT and Mel1c/PKC/ERK pathways in bursa. p-AKT: phosphorylated AKT; p-PKC: phosphorylated PKC; p-ERK1/2: phosphorylated ERK1/2; p-GSK-3*β*: phosphorylated GSK-3*β*.

In summary, a combination of green and blue monochromatic light not only promoted bursa B-lymphocyte proliferation and development on bursa histological structures but also increased melatonin secretion in chickens (Figure 10). Melatonin exerted its proliferation-inducing effect on LPS-stimulated B-lymphocytes by binding to the membrane receptors Mel1a and Mel1c with subsequent activation of the crosstalk between Gi/PI3K/AKT and PKC/ERK signals, leading to downstream stimulation of GSK-3*β* and *β*-catenin. In addition, melatonin may regulate a green and blue monochromatic light combination-induced B-lymphocyte proliferation by changing the antioxidative capacity. Our results provide new perspectives for the understanding of differential cellular responses upon Mel1a- or Mel1c-dependent activation of the same pathway (GSK-3*β*/*β*-catenin) by the same ligand (melatonin).

## Figures and Tables

**Table 1:** Light parameters.

Items	Light treatments						
	WW	RR	GG	BB	G→B	B→G	R→B
Light wavelength (nm) (1-26 days)	400-700	660	560	480	560	480	660
Light wavelength (nm) (27-42 days)	400-700	660	560	480	480	560	480
Light intensity (W/m^2^)	0.19	0.19	0.19	0.19	0.19	0.19	0.19
Photoperiod (light : dark)	23 : 1	23 : 1	23 : 1	23 : 1	23 : 1	23 : 1	23 : 1

**Table 2:** Sequences of primers used for RT-PCR.

Gene	Product size	Primer sequences (5′–3′)	Accession no.
*Mel1a*	333	F: CAATGGATGGAATCTGGGA R:GCTATGGGAAGTATGAAGTGG	NM_205362.1
*Mel1b*	259	F:TTTGCTGGGCACCTCTAAAC R: CGCTTGCTCTTCTGTCCATC	XM_417201.2
*Mel1c*	237	F:CACTATGGGAAACATTCACTGCC R:TATAGCAGCAGGTGTTCTTCAGG	NM_205361.1
*GAPDH*	124	F:ATCACAGCCACACAGAAGACG R:TGACTTTCCCCACAGCCTTA	NM_204305

F = forward primer; R = reverse primer.

## Data Availability

The data used to support the findings of this study are available from the corresponding author upon request.
